# Hjernetegn.dk—The Danish Central Nervous System Tumor Awareness Initiative Digital Decision Support Tool: Design and Implementation Report

**DOI:** 10.2196/58886

**Published:** 2024-07-25

**Authors:** Kathrine Synne Weile, René Mathiasen, Jeanette Falck Winther, Henrik Hasle, Louise Tram Henriksen

**Affiliations:** 1 Department of Pediatric and Adolescent Medicine Aarhus University Hospital Aarhus Denmark; 2 Department of Clinical Medicine Faculty of Health Aarhus University Aarhus Denmark; 3 Department of Pediatric and Adolescent Medicine Copenhagen University Hospital Copenhagen Denmark; 4 Department of Clinical Medicine Faculty of Medicine University of Copenhagen Copenhagen Denmark; 5 Danish Cancer Institute Danish Cancer Society Copenhagen Denmark

**Keywords:** digital health initiative, digital health initiatives, clinical decision support, decision support, decision support system, decision support systems, decision support tool, decision support tools, diagnostic delay, awareness initiative, pediatric neurology, pediatric neurology, pediatric CNS tumors, CNS tumor, CNS tumour, CNS tumours, co-creation, health systems and services, communication, central nervous system

## Abstract

**Background:**

Childhood tumors in the central nervous system (CNS) have longer diagnostic delays than other pediatric tumors. Vague presenting symptoms pose a challenge in the diagnostic process; it has been indicated that patients and parents may be hesitant to seek help, and health care professionals (HCPs) may lack awareness and knowledge about clinical presentation. To raise awareness among HCPs, the Danish CNS tumor awareness initiative hjernetegn.dk was launched.

**Objective:**

This study aims to present the learnings from designing and implementing a decision support tool for HCPs to reduce diagnostic delay in childhood CNS tumors. The aims also include decisions regarding strategies for dissemination and use of social media, and an evaluation of the digital impact 6 months after launch.

**Methods:**

The phases of developing and implementing the tool include participatory co-creation workshops, designing the website and digital platforms, and implementing a press and media strategy. The digital impact of hjernetegn.dk was evaluated through website analytics and social media engagement.

**Implementation (Results):**

hjernetegn.dk was launched in August 2023. The results after 6 months exceeded key performance indicators. The analysis showed a high number of website visitors and engagement, with a plateau reached 3 months after the initial launch. The LinkedIn campaign and Google Search strategy also generated a high number of impressions and clicks.

**Conclusions:**

The findings suggest that the initiative has been successfully integrated, raising awareness and providing a valuable tool for HCPs in diagnosing childhood CNS tumors. The study highlights the importance of interdisciplinary collaboration, co-creation, and ongoing community management, as well as broad dissemination strategies when introducing a digital support tool.

## Introduction

### Aim and Context

**Figure 1:**
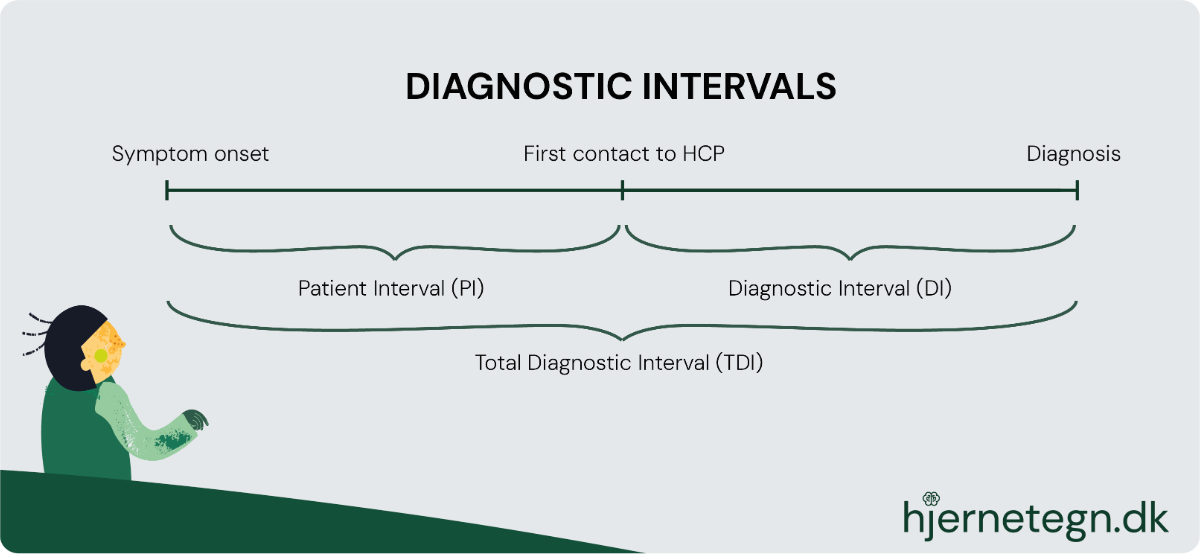
The diagnostic route: visualization of diagnostic intervals. HCP: health care professional.

Primary tumors of the central nervous system (CNS) are the second most prevalent childhood cancer, constituting approximately 20% of all cases [1,2]. The 5-year survival has risen to 75% [3], but survivors face severe late effects [4]. In Denmark, approximately 50 patients younger than 18 years are diagnosed every year [3]. Early diagnosis is crucial for the quality of life of survivors, and early detection and diagnostic delay have been in focus for decades [5-7]. The presenting symptoms at the time of onset can be vague, adding difficulty to diagnostics [8]. Time from first symptom to diagnosis can be divided into intervals, with the potential to identify inequities in the diagnostic process [9]. The total diagnostic interval (TDI) is the sum of the patient interval (PI) and the diagnostic interval [DI] PI: the time from symptom onset to first contact to a health care professional (HCP). DI: time from first contact to an HCP to diagnosis. Intervals are shown in Figure 1).

Over the past decade, research has reported TDI ranging from 28 to 123 days [10-26]. Recent studies indicate a knowledge gap in HCPs, causing a delay in the DI, and advocate for interventions specifically aimed toward HCPs to reduce the DI [27-29]. To map the trajectory for Danish patients, a questionnaire study was undertaken in 2022. The results showed an elongated median TDI of 106 days, with DI providing the larger part of the delay, thus displaying that challenging diagnostic processes are present in Denmark too. The results in detail have been reported separately [30].

**Figure 2:**
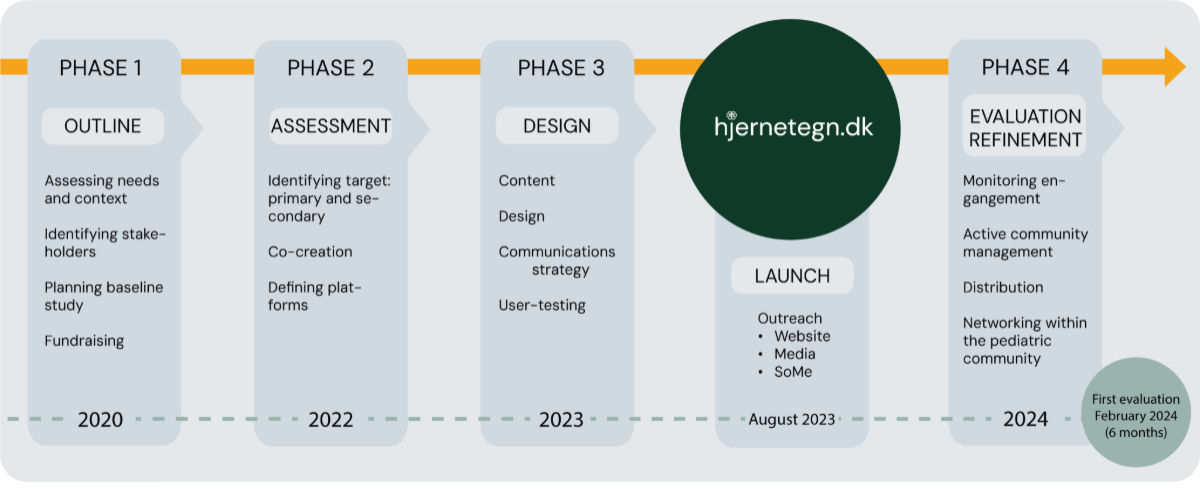
Workflow creating hjernetegn.dk. The initiative was launched on August 21, 2023. The evaluation of the digital impact was initiated in February 2024, 6 months after launch. SoMe: social media.

In 2017, The Danish Collaborative Comprehensive Childhood CNS Tumor Consortium (5C) was established, providing a long-term strategic research platform, to accelerate diagnostics and reduce late effects specifically in patients with CNS tumors [31]. The decision was made to create a Danish childhood CNS tumor awareness initiative, with the aim of reducing diagnostic delay. hjernetegn.dk was launched after 4 years of preparation. Figure 2 shows the phases of progression from 2020 onward.

### Similar Interventions

Efforts to accelerate timely diagnosis to enhance survival and the quality of life after cancer are advocated for worldwide. Childhood cancer has been a World Health Organization priority since 2018 [32]. In the United Kingdom, the HeadSmart campaign was initiated in 2011; actively reducing the time from symptom onset to diagnosis by several weeks, with a reduction in the time from onset until families approached a HCP, as well as an accelerated referral within the primary and secondary sector [18,33,34]. The HeadSmart initiative was set up as a nationwide public campaign, creating awareness in the public and among HCPs on alarm symptoms of CNS tumors in children.

Different organization of, and access to, the health care system may influence the differences in delay between countries. For hjernetegn.dk, we found inspiration in the methods of the HeadSmart campaign, but the Danish medical culture and health care organization, as well as being in an era of digitization, required designing a novel approach for the Danish initiative including constricting our target audience to HCPs.

The implementation is reported in accordance with iCHECK-DH (Guidelines for the Reporting on Digital Health Implementation) [35].

## Methods

We report the approach and implementation of the Danish CNS tumor initiative hjernetegn.dk, a tool to support the diagnostic process of childhood CNS tumors in the interface between primary and secondary care.

### Target

HCPs targeted for the initiative and tool were determined based on their involvement in clinical diagnostics, thus covering all HCPs who would first assess a child presenting with complaints that might be caused by a CNS tumor. Primary targets were general practitioners (GPs), ophthalmologists, and pediatricians. Secondary targets were nurse practitioners; neurologists; psychiatrists; ear, nose, and throat doctors; optometrists; child physiotherapists; and chiropractors.

We approached all specialists with a vested interest in contributing to the content and guidelines provided. We also included associations from specialties identified as recipients, involving them in the early process. National health authorities were informed prior to launch, timely enough for them to be able to respond but not with the intent to consult. Patient associations and advocacy groups were informed as well.

### Participating Entities

Setting up hjernetegn.dk required interdisciplinary experts, covering academic and clinical knowledge, communication strategy skills, and digital know-how. A qualified team of pediatric oncologists formulated the idea, nested within the 5C, and then included the Danish Cancer Society and the Danish Childhood Cancer Foundation in the collaboration to support the commercial and communications strategical part of the initiative.

### Hjernetegn.dk: Web Use, Website, and Social Media

We invited specialists from the primary target groups to participate in a co-creation workshop, encouraging “a collaborative approach of creative problem solving between stakeholders” [36]. The approach was set up to create a user journey map to identify the workflow in general practice and pediatric clinics, and to define what would be required in content and design to make the decision support tool feasible and successful.

The established key points were carried into the process of defining which platforms to make our tool accessible. In a postpandemic online era, printed material is obsolete in a Danish setting. To enable timely updated content, online platforms were used: a website as a nest for the tool and social media (SoMe) platforms to nest the outreach and network. We chose LinkedIn as the platform for SoME outreach based on its profile as a professional community for audience augmentation, with an algorithm that enables outreach by profession, favoring peer-to-peer communication [37]. Furthermore, funds were allocated to develop a LinkedIn campaign and ads on Google Search.

### Blueprint Summary: Hjernetegn.dk

The hjernetegn.dk website offers a list of alarm symptoms that require assessment to rule out a CNS tumor; a checklist for examination, listing what to include in the primary assessment; and finally, a decision support tool, offering a hands-on algorithm to decide whether to approach with watchful waiting, reevaluate within a certain timeframe, or refer directly for further evaluation including neuroimaging.

Content was provided by pediatric oncologists in collaboration with stakeholders from relevant specialties. Using national Danish guidelines and the National Institute of Health and Care Excellence–accredited Brain Pathways guideline [38] from the HeadSmart initiative as a backbone, website material applicable to a Danish setting was developed for the digital era, framing dissemination in a primarily digital strategy.

**Figure 3:**
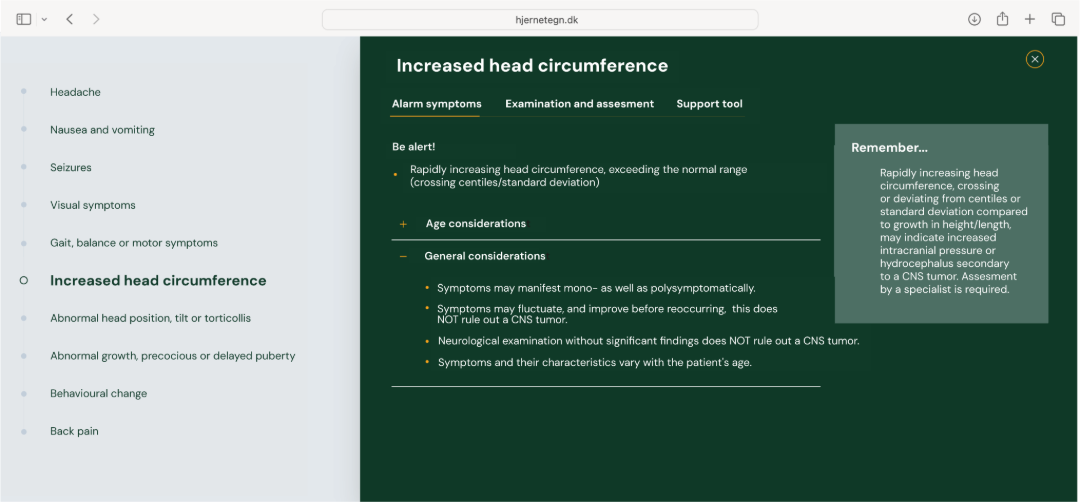
Alarm symptoms on hjernetegn.dk. CNS: central nervous system.

**Figure 4:**
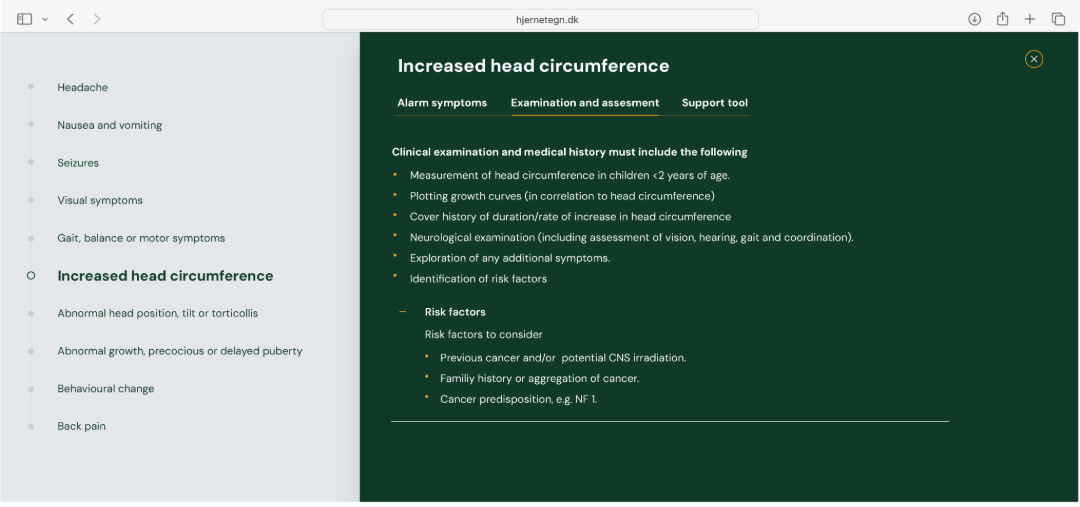
Examination and assessment on hjernetegn.dk. CNS: central nervous system.

**Figure 5:**
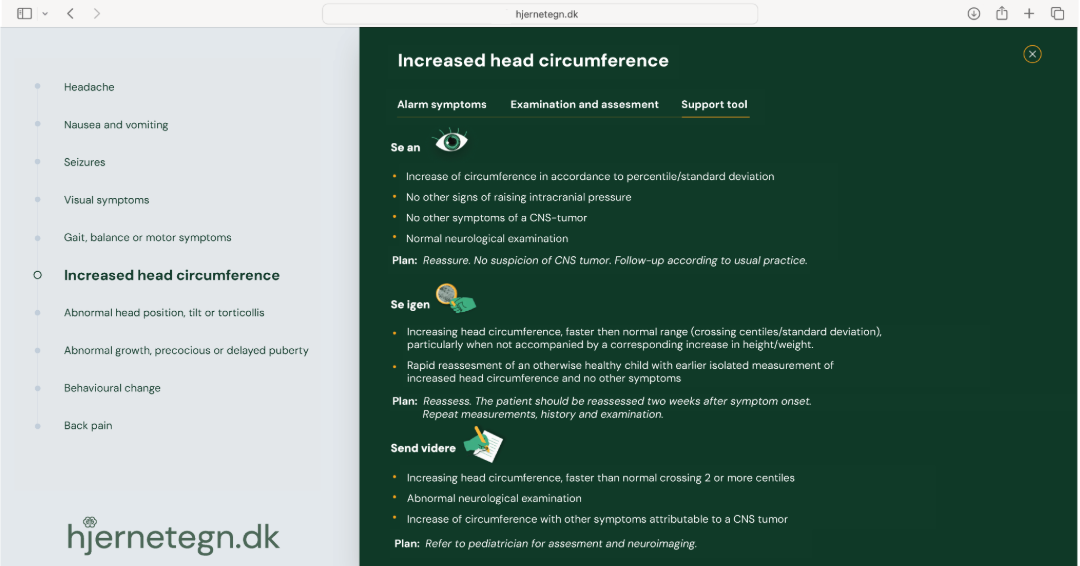
Support tool on hjernetegn.dk. CNS: central nervous system.

An example of the assessment tool is shown in Figures 3-5. Figure 3 displays alarm symptoms, Figure 4 displays points for examination and assessment, and Figure 5 displays the decision support tool.

A communications bureau provided design and graphical solutions.

The design accommodated the reported timeframe from the co-creation, allowing only 1 minute to unravel the algorithm. The aim was to facilitate an overview and to promote awareness of other symptoms to consider while using the tool for a specific assessment.

### Press/Media and Digital Strategy

The press/media strategy aimed to create awareness as well as drive users to the website when launched. Interviews were set up with major Danish health news outlets to be released on the day of launch. Within the first 3 months after release, 8 written articles were published in trade journals.

A written press release carrying information on the initiative and a graphical package was provided to all stakeholders, thereby inviting them to share the launch on their platforms and with their networks.

In the digital strategy, sponsored LinkedIn ads were planned for 3 iterations of advertising, each 3 weeks long (August 21, 2023, and 12 weeks after), and a Google Search strategy and ads first ran for 2 months, with plans to restrategize later. Furthermore, a plan for the organic content for the LinkedIn profile was designed. To embrace the algorithm, weekly posts were planned.

### Goals for Digital Performance the First 6 Months After Launch

We expected high activity at launch and in the following months, aligning with press releases and published articles. We presumed we would reach a more indicative plateau after 2 months.

Key performance indicators (KPIs), measurable variables for the evaluation of an initiative, were defined covering the number of visitors to the website, number of impressions on LinkedIn (impressions are defined as the number of exposures to posts or ads of individual feeds on the platform; organic impressions are defined as tailored content managed by hand), and number of searches on Google Search.

Defining the KPIs for using the website, we set a standard estimate of the possible number of assessments of children who visit their general practice, suggesting the number of cases where using the guideline and tool provided in hjernetegn.dk would be relevant. In consensus with experts from general practice, it was estimated that less than 20% of children’s visits with their GP require active use of guidelines, and by the same consensus, most likely <1 patient per week would require the GP to use hjernetegn.dk. With 3500 practicing doctors in Denmark, the KPI for visitors to the website was set to 500 visits per month. On LinkedIn, the KPI was set to 180,000 impressions, estimated from the number of possible recipients by the stated occupation on the platform. In Google Search, the KPI was set to 500 clicks per month, estimated from GP usage and searches. For the web page bounce rate KPI, the percentage of users departing from the entry page without further interaction was targeted as <55%.

### Data

Data analysis to monitor digital use and traffic was conducted utilizing Piwik PRO Analytics (version 16.26; Piwik Pro) [39]. Details on LinkedIn activity were extracted directly from the platform.

### Budget and Resources

The initiative was managed as part of a PhD study at Aarhus University Hospital, endorsed by the pediatric neuro-oncology network.

In the 4-year period, the allocation of work hours varied, with workload increasing and peaking at the time of launch. The project manager and a communications officer from the Danish Cancer Society had 20% of a full-time equivalent (FTE) workload from 2020 to 2022. Starting in 2023, the workload increased to 100% FTE for the project manager and 70% FTE for the communications officer.

Brand and graphic design, technical support, and digital solutions were provided by an external communications bureau. Collaboration and joint strategy were initiated 18 months before launch. The budget for the external bureau was approximately €100,000 (US $108,000), covering all external costs for building and implementing the initiative, as well as running the project 6 months after launch. Funding of approximately €65,000 (US $70,000) was allocated to refine, recommunicate, and manage the web page and SoMe platforms the second year after launch.

### Sustainability

The initiative is intended to become a part of a clinician’s toolbox, implying sustained use.

Implementation to stay is highly dependent on ongoing advocacy and a well-planned strategy for communication and up-to-date management of the community and web pages. Dissemination, through advertising, participating in relevant events, and reaching out on digital and real-life platforms are continuously required. Consequently, continuous funding is crucial.

Managing the community day to day requires active surveillance on SoMe channels to enable fast and sufficient responses, totaling 5 hours of work per week, inevitably spread out and not only as office hours.

### Ethical Considerations and Potential Barriers

It was important to balance the need to identify patients presenting strong indications for neuroimaging without instigating high numbers of unnecessary referrals and contacts to specialty units in both the primary and secondary sectors.

We acknowledged the risk and barrier that GPs might be reluctant to support the initiative due to worries of added workload in the primary sector.

Introducing an initiative as the one described, does not require ethical approval in the state of Denmark, when initiated by specialist networks as the 5C. Furthermore, when conducting the mentioned baseline studies [31], ethical approval was obtained from the Danish Data Protection Agency (file number: 1-16-02-300-19).

## Implementation (Results)

### Digital Results and Engagement

**Figure 6:**
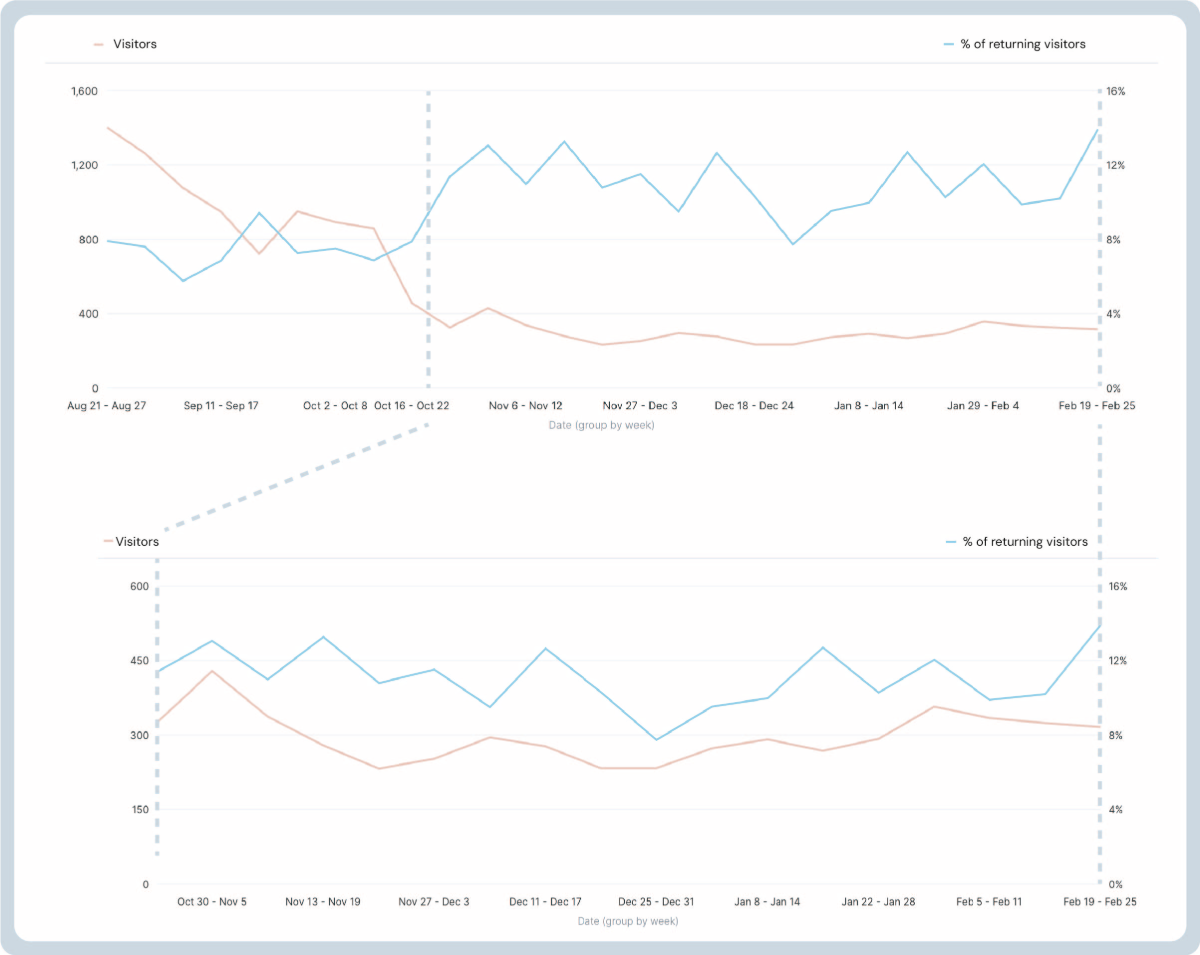
Website engagement 6 months after launch. Visitors on hjernetegn.dk from launch August 2023 to February 2024. The lower graph shows the number of visitors after the plateau was reached from November 2023 to February 2024.

hjernetegn.dk had 13,705 visitors from August 21, 2023, to February 25, 2024. Each visit had a mean of 6 engaging actions (click, scrolling, or other interactions). Figure 6 shows the number of visitors and returning visitors to the website in the first 6 months after launch. Access from smartphones and desktops covered 78% and 19%, respectively. The remaining 3% was accessed from other smart devices.

From August 21 to February 25 the overall bounce rate was 63%. On the page level, the bounce rate was <10%, indicating the use of the tool within the site. For desktop users, the bounce rate was 46%. The average time spent on the page was 1 minute and 45 seconds, and the average number of page views was 2.3.

By November, a plateau was met, showing an average of 300 visits per week, with a return rate of 10%. The bounce rate was reduced to 54% accordingly. The average time spent on a web page was 2 minutes, and the average number of web page views was 2.5 web pages.

On the website, 33,075 pages were viewed, with 60% (n=19,713 views, bounce rate 60%) of visits being the home page.

Half of all visitors accessed the web pages through Google Search, and 24% accessed them by URL. SoMe outlets constituted 21% of the visitors, particularly LinkedIn and Facebook. Less than 5% of visitors were directed by referral from other platforms, such as guidelines, health authority outlets, health care–related media, and collaborators’ websites.

**Figure 7:**
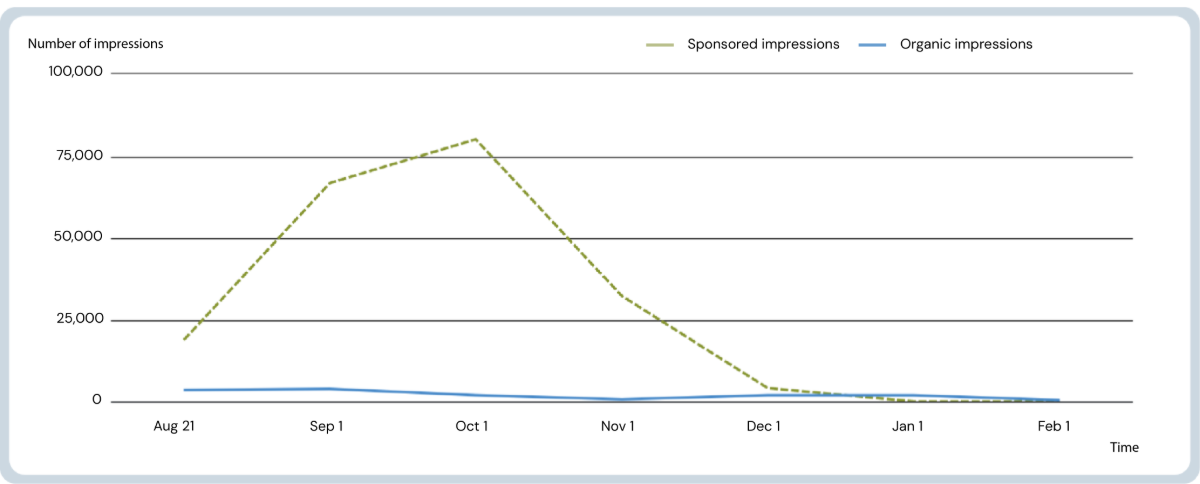
Impressions of active content on LinkedIn 6 months after launch. Impressions indicate number of exposures to individual user feeds. Sponsored impressions: impressions by ads. Organic impressions: tailored content managed directly from the hjernetegn.dk LinkedIn-page.

The sponsored LinkedIn ad campaign constituted the most impressions, covering 201,888 impressions and reaching the set KPI. Organic impressions formed 14,133 impressions in the same period. Figure 7 shows sponsored and organic impressions in the first 6 months.

### Implementation to Sustain

hjernetegn.dk is nested within the 5C and the four pediatric oncology centers nationwide. Efforts to maintain and update the website as well as strategizing to reach even more HCPs are ongoing. We are planning to solicit feedback from site users to better target HCPs and enhance the site and tool.

The hjernetegn.dk approach and guidelines are now taught in medical programs and among pediatric fellows as part of the curriculum, and the tool is continuously being introduced to GPs.

## Discussion

This study reports the development and implementation of hjernetegn.dk and the digital impact in the first 6 months after launch. The initiative covered the development of the digital diagnostic decision support tool and dissemination.

### Impact of the Digital Dissemination Strategy

Contemplating results from the digital strategy, as anticipated, we saw a very high interest following the main events around the launch, and from August 21 to October 22, the number of visitors peaked in alignment with publications in health media. From November, a plateau was reached, showing a stable number of visitors and returning visitors. When introducing new eHealth innovations, it is proposed in other works that the Gartner Hype Cycle can be applied [40,41]. It shows that reaching a plateau of productivity is natural but will be preceded by a so-called peak of inflated expectations and a trough of inflated expectations; although there was no true trough, the suggested phases in implementation matched our digital results, and the plateau was met with approximately 50 users daily, of whom 10% were returning visitors.

The results met our set KPIs. In fact, the number of visitors to the website far exceeded our expectations. At the plateau, the monthly number of visitors reached 1500, while the KPI was originally set to 500 within the first 6 months. This might have been a low target, but it was estimated based on expert notions of the number of contacts and that new guidelines take time to implement. Our results indicate that the communications strategy in our initiative has some effect. Using a so-called push-pull strategy, SoMe and ads endorsed and directed users to the website, introducing potential users to the tool, and the website as the actual tool had the effect of pulling in viewers. The bounce rate dropping over time indicates the tool is in actual use for supporting diagnostics and not only for visitors viewing the website.

For the LinkedIn campaign, the main focus was to create awareness and curiosity. A recent review on SoMe in public health campaigning presents the modified hierarchy of effect model, suggesting that health outcomes can be improved simply by SoMe exposure changing individuals’ behaviors [42]. Engagement leads to further impressions in secondary networks, raising awareness, directing traffic to the website, and supporting the push-pull strategy setup. Access from users via Facebook may be the result of user engagement on LinkedIn.

Moving forward, we will adjust set KPIs to encompass the results from the first 6 months of the initiative. Furthermore, it is being discussed whether to include more SoMe platforms.

### Lessons Learned

Inviting key stakeholders and primary targets to identify drivers and solutions and to point out barriers in the process is essential to design for sustainability. Finding a common understanding of the problem of diagnostic delay, especially when approaching GPs, was a time-consuming and delicate process. GPs were hesitant and implied that the motivation for the initiative was a criticism of their efforts in clinical practice, whereas the actual motivation was to provide support in a challenging process. The same issue was raised with the external communications consultants. A clear objective and common vocabulary is imperative for effective communication and collaboration between clinicians in different fields and the design producers. Barnes et al [43] advocate for interdisciplinary team approaches, bringing together specialists across the fields of health sciences, IT, and communications when producing initiatives in a computational health science era. We support this structural mindset as well as stress the importance of co-creation for content, setting the scene for successful health care interventions.

Through our efforts, we have strived to but not succeeded in reaching all GPs. In the interface between the analog and digital eras, substantial funds and resources are required to reach all areas. We realize some individuals will not be reached unless contacted directly.

The multiphase setup allowed stakeholders to participate continuously, which strengthened the implementation and is strongly advised. The design and platforms ensure flexible editing, allowing add ons easily. This enables future changes or revisions, or even more tools, to be embedded when needed.

When initiating a similar initiative, we would recommend the following:

- Establish a multidisciplinary team by involving communication experts, graphic designers, and technical designers from an early stage. Common ground and understanding of the aim during production is of utmost importance.
- Invite and engage stakeholders to define drivers for success and possible positive or negative attitudes toward the effort.
- Willingness to “kill your darlings.” As senders and clinical experts, be receptive to feedback from stakeholders if they do not see eye to eye.
- Have sufficient time and funding. Time is money, and fundraising takes time. In our case, it took 4 years from the formation of the project group to the launch of hjernetegn.dk. We had initially planned for 5 to 8 months.
- Develop realistic plans for follow-up and monitoring. Initiatives should be dynamic and easy to edit; create ongoing revision plans. If possible, conduct a baseline study so that both digital impact and impact in the clinic, as well as on the primary goal, namely the patients, can be measured and evaluated, and the initiative improved accordingly.

### Perspectives

We believe our study design is applicable to many rare diseases. Initiatives such as the website presented in this study create a hub for all applications necessary to recognize and interpret cardinal symptoms, followed by guidelines to support relevant and timely referrals. In this first edition, the initiative was targeted toward HCPs. In time, we will revisit the strategy and may plan to broaden the format to raise public awareness, thus covering the entire trajectory for patients with a CNS tumor.

## Abbreviations

5C: The Danish Collaborative Comprehensive Childhood CNS Tumor Consortium
CNS: central nervous system
DI: diagnostic interval
FTE: full-time equivalent
GP: general practitioner
HCP: health care professional
iCHECK-DH: Guidelines for the Reporting on Digital Health Implementation
KPI: key performance indicator
SoMe: social media
TDI: total diagnostic interval
